# Effect of Nurse-Led Intervention on Stress and Menstrual Parameters Regarding Menstrual Health Management Among Adolescent Girls

**DOI:** 10.7759/cureus.83478

**Published:** 2025-05-04

**Authors:** Smeeta S Revankar, Vaishali S Jadhav, Priya R Naik

**Affiliations:** 1 Obstetrics and Gynecological Nursing, College of Nursing, Bharati Vidyapeeth (Deemed to be University), Navi Mumbai, IND; 2 Medical Surgical Nursing, College of Nursing, Bharati Vidyapeeth (Deemed to be University), Navi Mumbai, IND

**Keywords:** adolescent girls, menstrual health management, menstrual parameters, nurse-led intervention, stress

## Abstract

Background

Research has revealed that numerous adolescent girls start menstruating without adequate information or readiness. Nevertheless, there is a lack of literature regarding the impact of educational interventions on improving the knowledge of adolescent girls in this regard. Thus, this study aimed to evaluate how a nurse-led intervention influences stress levels and menstrual parameters among adolescent girls in a chosen school in Navi Mumbai.

Methodology

The study employed a quantitative research approach and utilized a simple random sampling technique, specifically the lottery method, to select 80 (sample size calculated on the basis of the prevalence of a previous similar study) adolescent girls, with 40 in each group. Data collection involved the use of a self-structured questionnaire. Pre-hemoglobin levels for both the control and study groups were measured using a digital hemoglobin meter. A pre-test was conducted for both groups, focusing on demographic information, perceived stress, and menstrual health parameters. The study group received instruction in stretching exercises for alleviating dysmenorrhea, delivered by two nurse researchers, along with a booklet on menstrual parameters and daily distribution of two servings (100 g each) of iron-rich supplements in the form of laddoos.

Results

Analysis of the data utilized both descriptive and inferential statistics. The results indicated that the exercise intervention significantly reduced stress levels among adolescent girls in the study group regarding pain management during menstruation (mean=87.900, SD=8.53), with a high level of significance at p<0.001. Additionally, the changes in hemoglobin levels for both the control and study groups were 9.55 ± 2.1477 (p=0.058) and 10.225 ± 1.0975 (p=0.017), respectively, indicating a notable effectiveness of the nutritional supplement in improving anemia-related parameters in the study group. Furthermore, the distribution of booklets addressing menstrual parameters among adolescent girls in the study group resulted in a mean score of 6.20 ± 0.723, reflecting a positive change in menstrual health awareness and practices. The findings were extremely significant, with a p-value of less than 0.001, leading to the rejection of the null hypothesis H01.

Conclusion

The study suggests that replicating and implementing similar nurse-led educational interventions across all secondary schools in Navi Mumbai could systematically improve adolescents' understanding of menstruation and menstrual hygiene.

## Introduction

Nurses play a pivotal role as educators, leading interventions that follow a care delivery model encompassing assessment, evaluation, education, counseling, treatment, and other procedures through a holistic approach [1]. Menstrual health refers to a holistic state of well-being, encompassing physical, mental, and social dimensions rather than merely the absence of disease or illness within the context of the menstrual cycle [2]. In India, while menstruation is often regarded as a natural occurrence or even “a gift from God,” perceptions about it differ widely across various cultures and religions [3]. In many regions, limited awareness and numerous misconceptions about menstruation are commonly passed down from mothers to young girls. This often results in unnecessary fear, anxiety, and inappropriate practices among young girls and women. Research shows that menstrual knowledge and hygiene practices are also influenced by socio-economic status [4]. In rural areas, the lack of access to and inability to afford commercially available sanitary napkins have been linked to the use of old cloth, homemade pads, and cotton wool as alternatives [5].

Maintaining proper menstrual hygiene practices is essential for adolescent girls. This includes using sanitary pads, disposing of them appropriately, and ensuring proper hygiene by washing the genital area and hands. A lack of education and communication about these practices, along with reproductive health issues, exacerbates the challenge [6]. Consequently, increasing awareness about menstruation from childhood could promote safer practices and potentially alleviate the hardships faced by millions of women. Parik et al. [7] conducted a university-based descriptive cross-sectional study to assess the knowledge, attitudes, menstrual hygiene practices, and health-seeking behavior among adolescent girls, serving as a basis for future community interventions. The findings revealed that 79% of participants had adequate knowledge about menstruation, 82% experienced regular cycles, and 96% used sanitary pads. However, 74.8% refrained from visiting places of worship, 21% avoided physical activities, and 7.87% practiced social isolation during menstruation. Only 19% made dietary modifications. Common menstrual problems include dysmenorrhea, amenorrhea, menorrhagia, oligomenorrhea, premenstrual syndrome (PMS), premenstrual dysphoric disorder (PMDD), and irregular periods. These problems were found to be more prevalent among those with irregular cycles, infrequent absorbent changes, and inadequate cleaning practices. These issues can affect the regularity, duration, flow, or associated symptoms of menstruation. Although awareness existed, menstrual hygiene practices remained suboptimal, indicating a clear need for targeted education and intervention efforts [7].

In India, anaemia is the most prevalent nutritional concern [8], characterized by a hemoglobin level below 12 gm/dL. Adolescents are particularly vulnerable to anaemia due to rapid growth phases, menstruation, and inadequate dietary intake. Alarmingly, the prevalence of anaemia among adolescents remains high, with the National Family Health Survey 5 (NFHS-5) reporting that 59.1% of adolescent girls are affected [9]. Addressing anaemia can be both cost-effective and efficient through the provision of iron-rich diets. Iron deficiency anaemia (IDA) is the most prevalent form of nutritional anaemia worldwide. Adolescence marks the onset of menstruation in most girls, placing them at a higher risk of developing nutritional anaemia. To combat this condition, three key strategies are employed: supplementation, food fortification, and dietary diversification [10].

This intervention study aimed not only to provide access to menstrual products for adolescent girls in marginalized schools but also to educate them about best practices, the significance of an iron-rich diet, the role of exercise in alleviating menstrual pain, and stress management techniques for maintaining optimal menstrual health. Despite its inclusion in the Millennium Development Goals [10], menstrual hygiene remains a rarely addressed topic in school curricula. Therefore, it is crucial to examine existing misconceptions and malpractices related to menstrual knowledge, hygiene, dietary habits, and overall physical and mental well-being among adolescent girls. Understanding these issues is vital for designing future interventions to address them effectively.

Safe and effective menstrual health management serves as a catalyst for improved well-being and overall development among adolescent girls. Against this backdrop, this study was conducted to evaluate menstrual health parameters during menstruation, including stress levels affecting mental well-being, pain management, hygiene practices, and blood loss throughout the menstrual cycle.

The objectives of this study were as follows: to assess the pre-interventional menstrual health parameters in both the study and control groups; to evaluate the post-interventional menstrual health parameters in both groups; to compare the pre- and post-interventional menstrual health parameters within the study and control groups; to identify any associations between pre-interventional menstrual health parameters and selected demographic variables within the control group; and to examine the relationship between pre-interventional menstrual health parameters and selected demographic variables within the study group.

## Materials and methods

This quantitative research was conducted in a selected school in Navi Mumbai. The sample size was determined based on the prevalence reported in a similar study. The sample comprised 80 adolescent girls, with 40 in the study group and 40 in the control group. The study employed a randomized controlled trial (RCT) design, utilizing a probability simple random sampling technique, specifically the lottery method, for participant selection. Adolescent girls who were present during the data collection period, proficient in reading Marathi and English, had experienced menarche, and were aged between 12 and 15 years, studying in the seventh, eighth, and ninth grades, were included in the study. However, girls who were unwilling to participate or had not yet experienced menarche were excluded.

Approval for the study was obtained from the Institutional Ethical Committee (BV(DU)/CON/Navi Mumbai/EC/02/2022) and the Institute Research Recognition Committee. The investigator secured the necessary permissions from relevant authorities. Prior to participation, informed consent was obtained from parents, while assent forms were collected from the study participants.

Data collection procedure

Data collection involved the use of a self-structured questionnaire comprising three sections. Section A focused on socio-demographic variables, Section B covered the perceived stress scale, and Section C addressed menstrual health parameters, specifically menstrual hygiene and blood loss. Samples were selected based on predetermined inclusion and exclusion criteria. The tool underwent expert validation and was pretested to ensure its reliability. The test-retest method was applied to assess the reliability of the tool.

The pre-hemoglobin levels of both the control and study groups were assessed using a digital hemoglobin meter. A pre-test was administered using self-structured questionnaires. The study group received instruction in stretching exercises (conducted by two trained researchers) aimed at easing dysmenorrhea. Additionally, booklets were distributed to the study group, and study participants also received two daily servings (100 g each) of iron-rich supplements for a duration of five weeks.

The intervention included an exercise regimen consisting of a five-minute standing warm-up followed by 10 minutes of pelvic stretching exercises. Study group participants were engaged in this routine daily for five weeks, with each session lasting 15 minutes. A booklet was distributed to the study group, covering topics such as the significance of menstrual health, hygienic practices, dietary recommendations, exercise routines, mental health strategies, coping mechanisms, and self-care tips.

During the same five-week period, participants were provided with "ladoo" made from powdered dry dates, roasted groundnuts, and jaggery. Each ladoo contained 40 g of roasted groundnuts (providing 2.92 mg of iron and 1 g of protein), 40 g of dry dates (offering 1 mg of iron and 10.48 g of protein), and 20 g of jaggery (contributing 1 mg of iron and 0.16 g of protein). The nutritive value of 100 g of ladoo includes 4.92 mg of iron and 11.64 g of protein. Participants consumed two ladoos daily, totaling 9.84 mg of iron and 23.28 g of protein, under the supervision of the investigator. The acceptability of the prepared product was evaluated by a panel based on its color, taste, texture, and flavor. The researcher visited the school daily at a fixed time to distribute the ladoos and implement the exercise regimen. To ensure adherence, adolescents were instructed to consume the ladoos in the presence of the researcher.

After the five-week intervention period, a post-test was administered to both the control and study groups to assess changes in hemoglobin levels and perceived stress. Additionally, each participant of the control and study group received a complimentary pack of 10 sanitary pads each for four months. The booklet was also distributed to the control group after post-test data collection.

Data analysis was conducted using descriptive statistics for data processing. Hypotheses were evaluated using inferential statistics, paired t-tests applied.

## Results

Section 1

Demographic Variables of the Study and Control Groups

Table 1 presents the demographic variables of the control and study groups.

**Table 1:** Interpreting the demographic variables of the control and study groups

Demographic variables		Group			
		Control Group		Study Group	
		f	%	f	%
Age in years	12 years	8	20.0%	12	30.0%
	13 years	15	37.5%	21	52.5%
	14 years	14	35.0%	7	17.5%
	15 years	3	7.5%	0	0.0%
The class you study in	7th	3	7.5%	10	25.0%
	8th	16	40.0%	16	40.0%
	9th	21	52.5%	14	35.0%
Age of first menstrual period	10 years	1	2.5%	0	0.0%
	11 years	4	10.0%	8	20.0%
	12 years	19	47.5%	18	45.0%
	13 years	10	25.0%	11	27.5%
	14 years	6	15.0%	3	7.5%
Are your periods regular	No	16	40.0%	35	87.5%
	Yes	24	60.0%	5	12.5%
Religion	Hindu	35	87.5%	37	92.5%
	Christian	1	2.5%	2	5.0%
	Muslim	0	0.0%	1	2.5%
	Others	4	10.0%	0	0.0%
Area of residence	Urban	36	90.0%	34	85.0%
	Rural	4	10.0%	6	15.0%
Educational status of father	Illiterate	3	7.5%	0	0.0%
	Primary	13	32.5%	10	25.0%
	High School	15	37.5%	20	50.0%
	Higher Secondary	7	17.5%	8	20.0%
	Graduate	2	5.0%	2	5.0%
Educational status of mmother	Illiterate	5	12.5%	5	12.5%
	Primary	10	25.0%	13	32.5%
	High School	19	47.5%	18	45.0%
	Higher Secondary	6	15.0%	4	10.0%
Occupation of father	Employed	28	70.0%	33	82.5%
	Unemployed	3	7.5%	2	5.0%
	Self-employed	8	20.0%	5	12.5%
	Others	1	2.5%	0	0.0%
Occupation of mother	Employed	5	12.5%	6	15.0%
	Unemployed	19	47.5%	17	42.5%
	Self-employed	15	37.5%	13	32.5%
	Others	1	2.5%	4	10.0%
Number of siblings	1	24	60.0%	23	57.5%
	2	14	35.0%	16	40.0%
	3	0	0.0%	1	2.5%
	4	2	5.0%	0	0.0%
Source of information regarding menstrual health	Mother	37	92.5%	39	97.5%
	Friends	2	5.0%	0	0.0%
	Social Media	1	2.5%	1	2.5%
Monthly income	Rs. 5000-10000	12	30.0%	8	20.0%
	Rs 10001-15000	15	37.5%	11	27.5%
	Rs 15001-20000	5	12.5%	8	20.0%
	Rs 20001 and above	8	20.0%	13	32.5%
	Total	40	100.0%	40	100.0%

Section 2

Pre- and Post-interventional Stress Scores

Table 2 depicts the pre-test stress scores, showing that, in the control group, the majority (75%, n=30) had moderate stress, whereas, in the study group, 55% (n=22) had severe stress. None of the participants in either group had mild stress at baseline.

**Table 2:** Pre-test and post-test stress scores of the control and study groups Data are presented as number (percentage).

Control Group					
Levels	Categories of scores	Pretest Stress Scores		Post-test Stress Scores	
		f	%	f	%
Moderate	34-67	30	75	22	55
Severe	68-100	10	25	18	45
Study Group					
Moderate	34-67	22	55	37	92.50
Severe	68-100	18	45	3	8

Post-test results revealed that, in the control group, the majority (55%, n=22) had mild stress, while, in the study group, 92.5% exhibited mild stress levels. Notably, none of the participants in the study group had severe stress post-intervention.

Pre- and Post-test Hemoglobin Level

**Figure 1:**
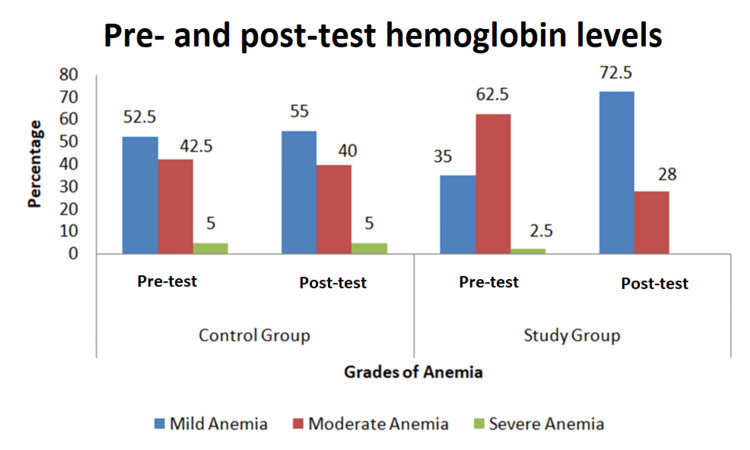
Distribution of adolescents on the basis of the severity of anaemia Data are presented as number (percentage).

Figure 1 interprets the pre- and post-test hemoglobin levels.

According to the World Health Organization, anemia in adolescent girls aged 9-15 years is defined as a hemoglobin level of <11.5 g/dL, with severity classified as follows: mild anemia: 11.0-11.4 g/dL, moderate anemia: 8.0-10.9 g/dL, and severe anemia: <8.0 g/dL [11].

In the current study, prior to the intervention, 21 (52.5%) adolescent girls in the control group and 14 (35%) in the study group were classified as having mild anemia. Following a five-week intervention involving the distribution of ladoos, 22 (55%) girls in the control group and 29 (72.5%) in the study group were found to have mild anemia.

Notably, there was a significant shift in the study group from moderate-to-mild anemia, with the proportion of girls with moderate anemia decreasing from 62.5% to 28% after the intervention. This suggests a positive impact of the nutritional intervention on improving hemoglobin levels.

Frequency and Percentage of Menstrual Health Parameters

Tables 3-4 present the frequency and percentage distribution of menstrual health parameters, focusing on hygiene and blood loss, within both the control and study groups. The findings indicate a shift in hygiene practices and a decrease in the duration of heavy bleeding days and overall menstrual flow length in the study group following the implementation of the stretching exercise.

**Table 3:** Menstrual health parameters - hygiene parameters Data are presented as number (percentage) n=40 each group.

S. No	Hygiene Parameters	Options	Control Group				Study Group			
			Pre-intervention		Post-intervention		Pre-intervention		Post-intervention	
			f	%	f	%	f	%	f	%
1	Nature of practice	Cloth	28	70	23	57.5	25	62.5	4	10
		Sanitary pads	12	30	17	42.5	15	37.5	36	90
2	Frequency of changing	3-4 hrs	3	7.5	10	25	3	7.5	30	75
		5-6 hrs	7	17.5	15	37.5	7	17.5	7	17.5
		7-8 hrs	30	75	5	12.5	32	80	3	7.5
3	Method of drying	Sunlight	12	30	18	45	15	37.5	4	10
		Corner of the room	0	0	0	0	2	5	0	0
		Toilet	16	40	5	12.5	8	20	0	0
		Not applicable	12	30	17	42.5	15	37.5	36	0.9
4	Method of washing cloth	Warm water & detergent	10	25	16	40	8	20	4	10
		Cold water &detergent	17	42.5	6	15	17	42.5	0	0
		Machine wash	1	2.5	1	2.5	0	0	0	0
		Not Applicable	12	30	17	42.5	15	37.5	36	0.9
5	Do you wash the perineal area after every urination/defecation	Yes	18	45	20	50	11	27.5	40	100
		No	19	47.5	20	50	26	65	0	0
		Not aware	3	7.5	0	0	3	7.5	0	0
6	Disposal of cloth/pad	Dustbin	15	37.5	22	55	12	30	30	75
		Pit	5	12.5	3	7.5	10	25	0	0
		Burn	10	25	5	12.5	10	25	4	10
		Any other-	10	25	10	25	8	20	6	15
		First, wash pads and discard them in the dustbin								
7	Months of usage of the same cloth	2 months	10	25	15	37.5	8	20	4	10
		3 months	9	22.5	8	20	0	0	0	
		4 months	7	17.5	0	0	7	17.5	0	
		≤ 5 months	2	5	0	0	10	20	0	
		Not applicable	12	30	17	42.5	15	37.5	36	90

**Table 4:** Menstrual health patameters- blood loss parameters Data are presented as number (percentage). n=40 each group

S. No	Blood loss parameters	Options	Control Group				Study Group			
			Pre-intervention		Post-intervention		Pre-intervention		Post-intervention	
			f	%	f	%	f	%	f	%
1	Duration of menstruation	3-4 days	15	37.5	12	30	10	25	26	65
		5-6 days	15	37.5	18	45	18	45	10	25
		≤ 7 days	10	25	10	25	12	30	4	1
2	Number of heavy bleeding days	1 day	15	37.5	18	45	10	25	18	45
		2 days	15	37.5	15	37.5	18	45	15	37.5
		≤ 3 days	10	25	7	17.5	12	30	7	17.5
3	Number of pads/cloth changed on heavy bleeding days	2.-3	15	37.5	19	47.5	8	20	17	42.5
		4.-5	12	30	13	32.5	20	14	19	47.5
		≤ 6	13	32.5	8	20	12	30	4	10
4	Number of pads/cloth changed on regular or routine blood flow	1-2	12	30	15	37.5	10	25	18	45
		3-4	16	40	16	40	15	37.5	14	13
		≤ 5	12	30	9	22.5	15	37.5	8	20
5	Last menstrual period normal in length and flow	Yes	18	45	19	47.5	15	37.5	32	80
		No	22	55	23	57.5	25	62.5	8	20
6	Type of pad/cloth used during heavy bleeding days	Regular	8	20	10	25	14	35	17	42.5
		Large	20	50	18	45	18	45	20	50
		Extra Large	12	30	12	30	10	25	3	7.5
7	Any previous history of anemia?	Yes	8	20	8	20	32	80	32	80
		No	32	80	32	8	8	20	8	20

Section 3

Effectiveness of Exercises to Reduce Stress 

Stress was assessed using self self-structured five-point stress scale on menstrual health parameters.

Table 5 presents the assessment of the effectiveness of exercises in reducing stress among the study group of adolescent girls regarding pain management during menstruation. The mean score of 87.900 with a standard deviation of 8.53 was highly significant, with a p-value of 0.079 at a significance level of less than 0.001. Consequently, the research hypothesis H01 was rejected.

**Table 5:** Effectiveness of exercises to reduce stress Data are presented as number (percentage), mean, and standard deviation. Paired t test (p-values of <0.01 were considered significant). n=40 each group

Group		Mean	Std. Deviation	t test	p value
Control group	Pre-stress Score	62.575	7.54096	-1.8	0.079
	Post-stress Score	66.55	13.54376		
Study group	Pre-stress Score	66.75	9.2895	-11.7	0
	Post-stress Score	87.9	8.5389		

Effectiveness of Nutritional Intervention on Hemoglobin

Table 6 displays the hemoglobin values of adolescent girls before supplementation (0 day), ranging from 6.5 to 13 g/dL, with a mean value of 9.425 and a standard deviation of 2.3082 in control subjects. In experimental subjects, the hemoglobin values at 0 day ranged from 6.5 to 10.9 g/dL with a mean of 9.925. The study evaluated the effectiveness of a five-week (35-day) nutritional intervention involving the daily distribution of nutrient-rich ladoos to adolescent girls in the study group. This intervention aimed to improve hemoglobin levels and reduce the prevalence and severity of anemia. The changes in both control and experimental subjects showed a mean of 9.55 with a standard deviation of 2.1477 (p=0.058) and 10.225 with a standard deviation of 1.0975 (p=0.017), respectively. These results indicate the notable effectiveness of the nutritional supplement in improving hemoglobin levels and reducing the severity of anemia among adolescent girls, especially in the study group, leading to the rejection of the research hypothesis H01.

**Table 6:** Effectiveness of nutritional intervention on hemoglobin Data are presented as number (percentage), mean, and standard deviation. Paired t test (p-values of <0.01 were considered significant). n=40 each group; Hb=haemoglobin

Group		Mean	Std. Deviation	Paired t test	p value	
Control group	Pre-HB Value	9.425	2.3082	-1.955		
	Post-HB Value	9.55	2.1477		0.058	
Study group	Pre-HB value	9.925	1.1633	-2.504		
	Post-HB Value	10.225	1.0975		0.017	

Effectiveness of the Booklet on Menstrual Parameters

Table 7 provides an analysis of the effectiveness of the booklet on menstrual parameters among the study group of adolescent girls. The mean score of 6.20 with a standard deviation of 0.723 was highly significant, with a p-value of 0.65 at a significance level of less than 0.001. As a result, the research hypothesis H01 was rejected.

**Table 7:** Effectiveness of the booklet on menstrual parameters Data are presented as number (percentage), mean, and standard deviation. Paired t test (p-values of <0.01 were considered significant). n=40 each group

Group		Mean	Std. Deviation	t test	p value
Control group	Pre-menstrual Parameter Score	3.9	1.336	-0.458	0.65
	Post-menstrual Parameter Score	4.03	1.143		
Study group	Pre-menstrual Parameter Score	4.4	1.297	-8.35	0
	Post-menstrual Parameter Score	6.2	0.723		

Section 4

Association Between Pre-interventional Parameters and Selected Demographic Variables in the Control Group

Table 8 presents the results of the Pearson test aimed at determining any potential association between stress levels and selected demographic variables. The calculated chi-square value for the demographic variable, educational status of the mother, was significantly associated with pre-interventional knowledge score in the control group at a significance level of 0.05. Therefore, hypothesis H02 is accepted for educational status of the mother and rejected for all other selected demographic variables.

**Table 8:** Association between pre-interventional parameters and selected demographic variables in the control group Data are presented as number (percentage). Chi-square test (p-values of <0.05 were considered significant).

	Association of demographic variable with pre-stress	df			Whether significant at the 0.05 level
Sr. No			Chi-square	p	
			Value	Value	
1			Age in years	3		7.050	0.070	Non-significant
2	Class you study	2	2.070	0.355	Non-significant
3	Age of first menstrual period	4	7.956	0.093	Non-significant
4	Are periods regular	1	0.029	0.865	Non-significant
5	Religion	2	0.371	0.831	Non-significant
6	Area of residence	1	0.943	0.332	Non-significant
7	Educational status of father	4	5.401	0.249	Non-significant
8	Educational status of mother	3	12.428	0.006	Significant
9	Occupation of father	3	0.874	0.832	Non-significant
10	Occupation of mother	3	5.101	0.165	Non-significant
11	Number of siblings	2	5.657	0.059	Non-significant
12	Source of information	2	0.688	0.709	Non-significant
13	Monthly income	3	3.290	0.349	Non-significant

Association Between Pre-interventional Parameters and Selected Demographic Variables in the Study Group

Table 9 demonstrates that the calculated chi-square value for the demographic variable monthly income is significantly associated with pre-interventional knowledge scores, at a significance level of 0.05 in the study group. Consequently, hypothesis H02 is accepted for monthly income and rejected for other demographic variables.

**Table 9:** Association between pre-interventional parameters and selected demographic variables in the study group Data are presented as number (percentage), mean, and standard deviation. Chi-square test (p-values of <0.05 were considered significant).

	Association of demographic variable with pre-stress	df			Whether significant at the 0.05 level
Sr. No			Chi-square	p	
			Value	Value	
1	Age in years	2	1.905	0.386	Non-significant
2	Class you study	2	3.158	0.206	Non-significant
3	Age of first menstrual period	3	.978	0.807	Non-significant
4	Are periods regular	1	2.707	0.100	Non-significant
5	Religion	2	0.171	0.918	Non-significant
6	Area of residence	1	0.372	0.542	Non-significant
7	Educational status of father	3	1.053	0.789	Non-significant
8	Educational status of mother	3	2.573	0.462	Non-significant
9	Occupation of father	2	0.447	0.800	Non-significant
10	Occupation of mother	3	0.753	0.861	Non-significant
11	Number of siblings	2	1.556	0.459	Non-significant
12	Source of information	1	0.054	0.816	Non-significant
13	Monthly income	3	8.421	0.038	Significant

## Discussion

The findings of the study were discussed in relation to the objectives, hypotheses, and results. The present research demonstrated a significant reduction in stress related to menstrual pain among adolescent girls in the exercise study group (mean=87.900, SD=8.53), with statistical significance observed at the p<0.001 level.

The research findings were supported by Talekar et al. [12], who examined the impact of stretching exercises on menstrual pain among adolescent girls from selected schools in Navi Mumbai in 2021. Following the intervention, participants reduced their average pretest score from 2.65 to 1.99 in the post-test assessment. Moreover, post-test menstrual symptoms were significantly lower compared to pretest levels after eight weeks of intervention, providing evidence that stretching exercises contributed to alleviating menstrual pain and enhancing coping mechanisms (p-value<0.0001) [13]. In 2018, Dehnavi et al. [14] conducted a clinical trial study on the effect of aerobic exercise on primary dysmenorrhea. Participants were randomly assigned to intervention and control groups. A visual pain questionnaire was used as the instrument and was completed by both groups during the first three days of their menstrual cycle. The exercise protocol for the intervention group consisted of aerobic exercise performed three times a week for 30 minutes over a period of eight weeks. Data were analyzed using Fisher's exact test and the chi-square test. After four weeks, no significant changes were observed in the intervention group compared to the control group (p=0.423). However, at the end of eight weeks, the intervention group showed significant improvement compared to the control group (p=0.041) [13].

In the study group, 62.5% (25) of the girls used cloth during their periods, while 37.5% (15) used sanitary pads. Around 75% (30) of them changed their pads only after seven hours. When it came to disposal, 37.5% (15) threw the pads in dustbins, and 25% (10) washed them before throwing them away. Additionally, 47.5% (19) of the girls did not wash their private area after urinating, and 20% (eight) reused the same cloth for two to four months during their periods.

The findings of this study were supported by the research of Deshpande et al. [13], who investigated menstrual hygiene practices among adolescent girls in an urban slum area in Karad, Maharashtra, in 2017. Among the 100 participants, 60% used sanitary pads, approximately 19% relied on old household cotton, 16% purchased new cloth from the market, and only 5% used both pads and cloth. Among those using sanitary pads, 63.34% changed them frequently (>3 times/day). Additionally, about 70% washed the same cloth and reused it more than three times. Notably, 51.67% of the girls wrapped sanitary pads in paper before disposal, but an unconventional practice of washing sanitary pads before disposing of them in a carry bag was observed in this study, with around 30% of girls adopting this method. Furthermore, 18.4% of participants disposed of their pads openly [14]. In an epidemiologic study conducted to explore the knowledge, practices, and sources of information regarding menstruation and hygiene among adolescent girls in Bangalore, India, Ramachandra et al. [15] used a cross-sectional study method for 550 school-going adolescent girls aged 13-16 years. Around 34% of the participants were aware of menstruation prior to menarche, and mothers were the main source of information among both groups. Overall, 69% of adolescent girls were using sanitary napkins as menstrual absorbents, while 6% were using both cloth and sanitary napkins. Almost half of the rural participants dried the absorbent inside their homes [15].

The results of the present study indicated that the effectiveness of the booklet regarding menstrual health management during the menstrual cycle (mean=6.20, SD=0.723) was highly significant, with a p-value <0.001. A significant difference was also observed between the post-interventional parameters in the study and control groups.

These findings were reinforced by the work of Kalabarathi et al. [16], who conducted a quasi-experimental study in 2019 to evaluate the effectiveness of an informational booklet on menstrual hygiene knowledge among higher secondary school girls in Tamil Nadu. The study involved 50 participants. The pretest mean score of 8.94 (SD=1.39) increased to a post-test mean score of 18.22 (SD=1.46), reflecting a mean difference of 9.28. The paired t-value of 35.8594 and a p-value of <0.05 indicate a statistically significant improvement. These results demonstrate the effectiveness of the booklet in enhancing menstrual hygiene knowledge among higher secondary school girls, which may contribute to improved menstrual hygiene practices [16]. A study was conducted to evaluate the effectiveness of a pamphlet on menstrual hygiene management among adolescent girls in a selected school [17]. A pre-test was administered using structured knowledge questionnaires, and, on the same day, the pamphlet was distributed. Comparisons between the pre-test and post-test knowledge scores were made using a paired t-test, conducted at a 5% level of significance. The pre-test average score was 8.11 with a standard deviation of 2.15, while the post-test average score was 18.26 with a standard deviation of 1.36. The paired t-test yielded a test statistic value of 42.35, with a p-value of 0.00, which was less than 0.05. The study demonstrated that the pamphlet was effective in improving knowledge regarding menstrual hygiene management among adolescent girls in the selected school [17].

In the present study, a significant association was observed between the use of the nutritional supplement and improved outcomes among anaemic adolescent girls in the study group, indicating its potential efficacy (post Hb mean=10.225, SD=1.0975) at a p-value of <0.001.

Dhiman et al. [18] conducted an experimental study in 2017 to evaluate the effectiveness of nutritional interventions on hemoglobin levels among anaemic adolescent girls. The study was conducted in two rural schools selected through simple random sampling from six rural schools in Rajpura Tehsil, Patiala District, Punjab. The results indicated a high prevalence of anaemia among adolescent girls (at 95.5%). Following the implementation of nutritional interventions, the mean post-hemoglobin level in the experimental group was 9.47, compared to 8.77 in the control group, resulting in a mean difference of 0.70. The calculated t-value of 2.24 was statistically significant at the 0.05 level [18]. Mathubala et al. [19] conducted a study to assess the effectiveness of an iron supplementary ball on haemoglobin levels among adolescent girls with nutritional deficiency anaemia at selected nursing colleges in Thanjavur. The investigator measured haemoglobin levels in both the experimental and control groups using Sahli’s method [19]. From day two to day 31, the experimental group received the iron supplementary ball intervention after breakfast, administered under the supervision of the researcher for a duration of 30 days. After the intervention period, the post-test haemoglobin levels were assessed in both groups. The experimental group had a mean haemoglobin level of 10.27 with a standard deviation of 1.91, which was higher than the control group’s mean of 9.49 with a standard deviation of 1.38. The calculated t-test value was 10.933, which exceeded the table value of 2.05 (CV > TV), indicating statistical significance at the 0.05 level. Therefore, the study concluded that the iron supplementary ball was effective in improving haemoglobin levels among adolescent girls [19].

In the present study, the chi-square test was employed to examine potential associations between stress levels and selected demographic variables. The findings indicated that, in both the control and study groups, the educational status of the mother and monthly income were below the table value at the 0.05 level of significance.

These results were corroborated by Komalavalli et al.’s [20] study conducted in 2016, which investigated the effectiveness of a structured teaching program on knowledge and practices related to sanitary napkins among schoolgirls in Kancheepuram district. The chi-square analysis revealed a statistically significant association between demographic variables such as the occupational status of the father and the frequency of changing napkins and the level of knowledge concerning sanitary napkins among schoolgirls, with a significance level of p < 0.05 [20].

Limitations

The study included only 40 participants in each group (control and intervention), which, despite revealing a significant difference between the groups, may limit the extent to which the findings can be generalized to a broader population. The intervention was conducted over a period of just five weeks, which may not be sufficient to observe long-term effects on stress levels and menstrual health outcomes. A duration of five weeks for the dietary supplementation may be insufficient to produce a substantial impact on anaemia.

The study was conducted in a single school, so the results did not reflect the diverse cultural, social, and economic backgrounds of adolescent girls in other regions. Factors such as diet, physical activity, and emotional support from family or peers, which could affect stress and menstrual health, were not controlled.

## Conclusions

Menstrual pain emerged as a prevalent issue among adolescent girls in schools across Navi Mumbai, Maharashtra, India, significantly affecting school attendance. Regular exercise offers a promising, accessible, and cost-effective strategy to alleviate dysmenorrhea, although its effectiveness may depend on the quality, intensity, and duration of physical activity. Additionally, dry date powder and roasted groundnuts, being rich sources of iron and protein, show potential as natural dietary supplements for preventing and managing iron deficiency anaemia among adolescent girls.

The findings highlight the need for integrated, school-based health interventions that combine physical activity with affordable nutritional strategies to support adolescent girls' health and educational outcomes. Future research should explore long-term effects of such interventions, optimize exercise regimens for menstrual pain relief, and evaluate the sustained impact of iron-rich dietary supplements in larger and more diverse populations. Expanding the scope and duration of studies could provide deeper insights and more robust evidence to guide public health policies and practices targeting adolescent health.
